# Superwettable‐Membrane‐Assisted Extraction and Separation as a General Method for Removal of Organic Pollutants From Water

**DOI:** 10.1002/advs.202515526

**Published:** 2025-10-06

**Authors:** Deqi Wang, Ting Zhang, Fan Min, Yifeng Gao, Jiaming Zhu, Ganhua Xie, Zonglin Chu

**Affiliations:** ^1^ Key Laboratory of Chemo and Biosensing College of Chemistry and Chemical Engineering Hunan University Changsha 410082 P. R. China; ^2^ College of Chemistry and Chemical Engineering Shangqiu Normal University Shangqiu 476000 P.R. China; ^3^ Greater Bay Area Institute for Innovation Hunan University Guangzhou 511300 P. R. China

**Keywords:** anti‐fouling, extraction and separation, organic pollutants, superhydrophilic

## Abstract

Freshwater scarcity has emerged as a critical global challenge, driven by widespread water contamination from organic pollutants. Consequently, the development of simple, large‐scale, and environmentally benign treatment technologies for eliminating the organic pollutants is highly desirable. Here, an integrated in situ extraction approach coupled with superwettable membrane separation is introduced, enabling efficient and streamlined removal of organic pollutants from water. After being treated with this novel technology, the residual organic pollutants are < 1.5 ppm with a removal efficiency > 95.5% and a total organic carbon (TOC) < 40 ppm. By circumventing the operational complexity and contamination risks associated with conventional methods, the strategy offers a promising and scalable solution for advanced wastewater treatment.

## Graphical abstract

An integrated liquid‐liquid extraction and in situ superwettable membrane separation system is developed for efficient removal of organic pollutants from wastewater. After being treated with this novel technology, the residual organic pollutants are < 1.5 ppm with a removal efficiency > 95.5% and a TOC < 40 ppm, demonstrating a scalable and sustainable solution for the purification of organic pollutants‐contaminated water.

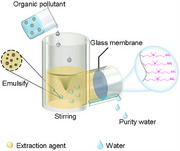

## 1 Introduction

Industrial chemicals, pesticides, and pharmaceuticals, originating from industrial, agricultural, and domestic activities, have been contributing to an escalating problem of organic pollutants in water resources.^[^ ^1^, ^2^, ^3^, ^4^, ^5^ ^]^ Characterized by low volatility and strong resistance to degradation due to their complex molecular structures, these contaminants pose significant challenges for removal using conventional treatment processes.^[^ ^6^, ^7^ ^]^ As a result, wastewater treatment facilities often fail to fully eliminate such pollutants, which remain widely detected in rivers, lakes, and soils.^[^ ^2^, ^8^, ^9^ ^]^ Developing efficient and scalable purification technologies is therefore imperative for mitigating organic pollution and safeguarding water quality.

A wide range of treatment strategies has been developed for the removal of organic pollutants, including advanced oxidation processes,^[^ ^10^, ^11^, ^12^ ^]^ biodegradation,^[^ ^13^ ^]^ membrane separation,^[^ ^14^, ^15^ ^]^ adsorption,^[^ ^1^, ^16^, ^17^ ^]^ liquid‐liquid extraction.^[^ ^18^, ^19^, ^20^ ^]^ Despite their utility, these approaches often face inherent limitations, such as operational complexity, long processing times, and poor reusability.^[^ ^21^, ^22^ ^]^ Fenton and Fenton‐like technologies, for example, can rapidly degrade organic pollutants through strong oxidizing radicals and are operationally straightforward. However, their application is constrained by issues of secondary pollution and catalyst deactivation.^[^ ^23^, ^24^ ^]^ Photocatalysis, while environmentally friendly and potentially effective, is hindered by its dependence on light conditions, high material costs, and challenges in catalyst recovery.^[^ ^21^, ^25^ ^]^ β‐cyclodextrin‐based adsorption materials offer strong affinity toward organic pollutants via host‐guest interaction,^[^ ^1^, ^17^, ^26^, ^27^ ^]^ but their limited cavity volume leads to rapid saturation and reduced long‐term efficiency. Liquid‐liquid extraction, though energy‐efficient and well‐suited to complex water matrices,^[^ ^20^ ^]^ typically requires cumbersome steps including vigorous mixing and static separation,^[^ ^28^ ^]^ and often results in residual extractants in the treated water, posing secondary pollution risks.^[^ ^29^ ^]^

To address these limitations, membrane‐based separation technologies have emerged as a promising alternative.^[^ ^30^, ^31^, ^32^, ^33^, ^34^ ^]^ Notably, membranes superwettability—especially those with superhydrophilic/underwater superoleophobic (SHUSO) properties— has been successfully employed in diverse liquid‐liquid separation scenarios.^[^ ^35^, ^36^, ^37^, ^38^ ^]^ During continuous oil‐water separation, oil droplets tend to adhere and accumulate on the porous surface of the membrane, leading to severe oil fouling and a significant reduction in separation capacity.^[^ ^39^, ^40^ ^]^ It has been proven that surface modification with strongly hydrophilic substances can effectively enhance the membrane's resistance to oil contamination.^[^ ^39^, ^41^, ^42^, ^43^ ^]^ Particularly, surface modification with zwitterionic substances such as sulfobetaine is preferred because of the coexistence of cationic and anionic groups within its molecular structure, both of which strongly binds water molecules through electrostatic interactions, enabling the formation of a dense hydration layer on the pore surface.^[^ ^44^, ^45^ ^]^ Therefore, sulfobetaine‐modified membranes permit efficient water transport while resisting fouling from organic phases, enabling high‐performance separation under continuous flow conditions.^[^ ^46^, ^47^ ^]^

Here, we present a novel, integrated approach for the continuous in situ removal of organic pollutants, combining liquid‐liquid extraction with SHUSO porous glass membranes. The membranes are fabricated by covalent attachment of sulfobetaine molecules onto silicone nanofilament‐coated glass substrates via thiol‐ene click chemistry, resulting in a surface with remarkable superhydrophilicity and underwater superoleophobicity (underwater oil contact angle (*θ*_OW_) > 160° and underwater oil sliding angle (*θ*_OSW_) < 5°). This system enables continuous extraction and efficient separation of a wide range of organic pollutants, achieving high removal efficiencies while minimizing residual extractants. This strategy represents a significant advancement in membrane‐assisted water purification and holds strong potential for real‐world environmental applications.

## 2 Results

### 2.1 Fabrication and Characterization of Glass Membranes

**Figure 1:**
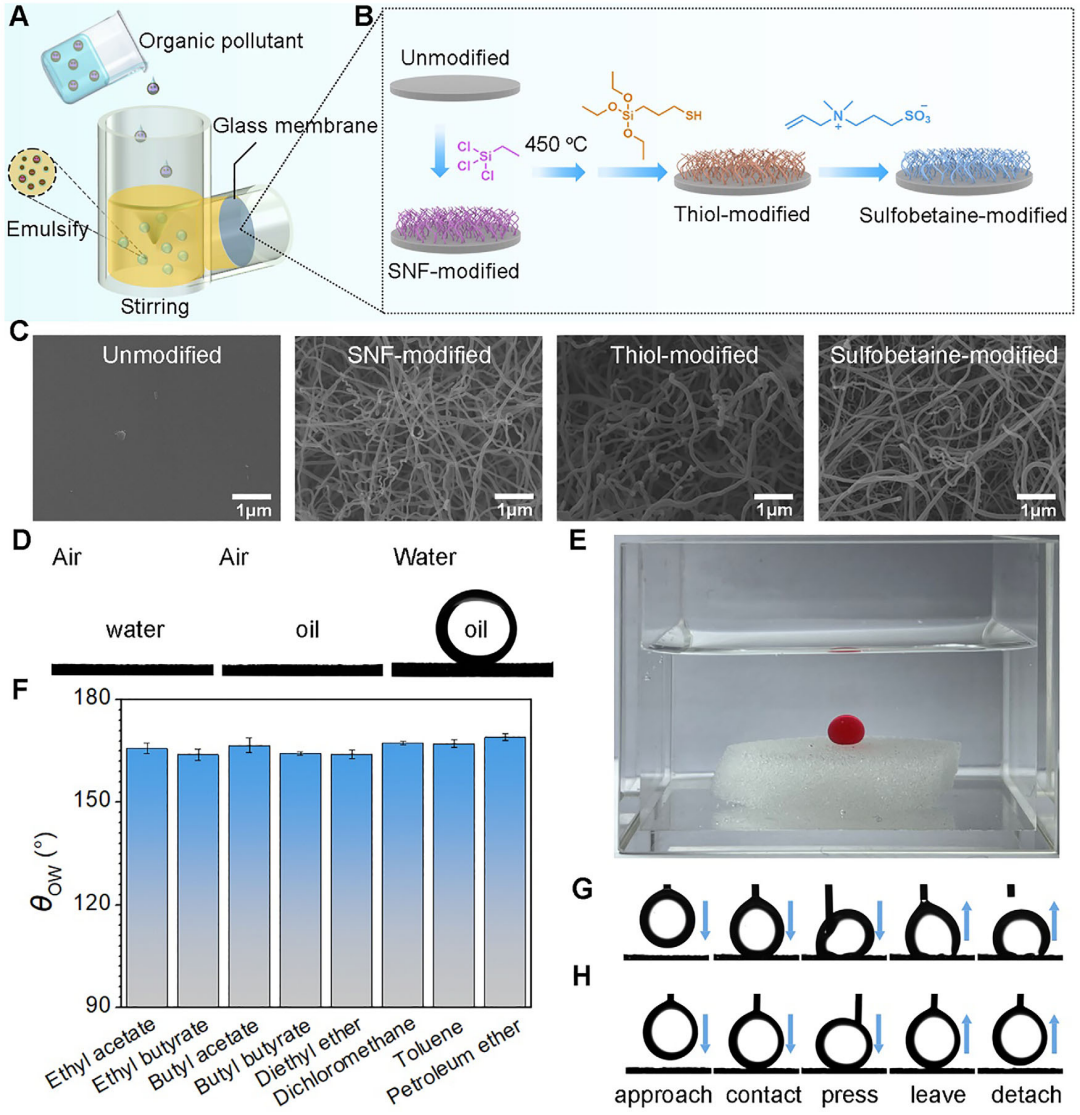
Fabrication and Characterization of the SHUSO Membrane. A) Schematic diagram of the SHUSO membrane system for the removal of organic pollutants from water samples. B) Process for the preparation of sulfobetaine‐modified glass membrane via a stepwise modification method. C) SEM images of glass membranes at different stages. D) Water contact angle in air (*θ*_W_), oil contact angle in air (*θ*_O_), and underwater oil contact angle (*θ*_OW_) for sulfobetaine‐modified G2 glass membrane. E) The image of an underwater oil droplet (1,2‐dichloroethane was dyed with oil red O) on the surface of sulfobetaine‐modified G2 glass membrane. F) *θ*_OW_ for various oils on the sulfobetaine‐modified G2 glass membrane. G) Dynamic oil‐adhesion tests on the unmodified G2 glass membrane surface, oil = 1,2‐dichloroethane. H) Dynamic oil‐adhesion tests on the sulfobetaine‐modified G2 glass membrane surface, oil = 1,2‐dichloroethane.

A continuous in situ extraction and separation device was developed by integrating of a porous glass membrane with a liquid–liquid extractor (**Figure** **1**A). The membrane, fabricated from sintered glass sand particles, featured a diameter of 3.6 cm. Given the propensity of membranes to suffer from oil fouling, a surface modification strategy was implemented to impart robust anti‐fouling properties through the introduction of sulfobetaine‐based superwettability. As shown in Figure 1B, the surface of the glass membrane was modified through a stepwise functionalization process. Firstly, a silicon nanofilaments (SNFs) network layer was deposited onto the surface of the glass membrane via chemical vapor deposition using ethyltrichlorosilane (ETCS) as a precursor. To introduce surface reactivity, the surface ethyl groups of the SNFs were oxidized into hydroxyl groups by heating at 450 °C in air. Subsequent functionalization was performed using (3‐mercaptopropyl)triethoxysilane, introducing thiol groups for further modification. Finally, vinyl‐functionalized sulfobetaine molecules were covalently attached onto the SNF‐coated surface via thiol‐ene click reaction, yielding a membrane with SHUSO properties. The surface morphologies of glass membranes were characterized by scanning electron microscopy (SEM), revealing the formation of a thin, uniform nanofilament network coating the sand particles after modification (Figure 1C). Elemental mapping via energy‐dispersive X‐ray spectroscopy (EDS) confirmed the presence and homogeneous distribution of key elements—C, O, Si, N, and S—associated with the sulfobetaine functional groups (Figure S1, Supporting Information), verifying successful and uniform surface modification.

It is worth noting that the pristine G2 glass filter (sintered glass filter made of sand microparticles) used in this work is a common type of glass filter, which is usually used for filtering large sediment particles and gas scrubbing. The pore size of such a G2 glass filter is 30−50 µm, according to the data provided by the manufacture. In order to show how the surface topography changes before and after the introduction of the nanofilament network clearly, high magnifications of SEM images are given in Figure 1C. In such high magnification images, only a small area of a sand microparticle is displayed in each image, and the pores between the sand microparticles cannot be seen. In order to show the pores between the sand microparticles, low magnification SEM images of the G2 glass filter before and after surface medication are provided in Figure S2 (Supporting Information). As shown, the pore size of the membrane before and after modification remains almost unchanged since there is only a thin layer of highly porous nanofilament network formed on the surface of the sand microparticles. In addition, we also found that the change in water flux of the membrane after surface modification is negligible (Figure S3, Supporting Information; as compared to the unmodified membrane), implying that the pore size of the membrane maintains almost unchanged before and after surface modification. Such a result is consistent with the SEM observations.

X‐ray photoelectron spectroscopy (XPS) was utilized to further confirm the successful surface modification of the glass membrane with sulfobetaine groups. C 1s (≈284 eV), O 1s (≈531 eV), Si 2s (≈154 eV), and Si 2p (≈103 eV) peaks can be detected from the surface of the glass membrane. With the introduction of the sulfobetaine group, obvious peaks of S 2p (≈168 eV) and N 1s (≈402 eV) appear in the spectrum of the glass membrane (Figure S4, Supporting Information). The emerging N 1s peak at ≈402 eV corresponds to the quaternary ammonium (C─N^+^) (Figure S5, Supporting Information). The high‐resolution spectrum of S 2p has two distinct sulfur peaks—the peak at ≈163.8 eV is attributed to the S─C bond, and the other peak at ≈168 eV can be assigned to the sulfonate group (─SO_3_^−^) (Figure S6, Supporting Information). These results indicate that sulfobetaine was successfully modified onto the surface of the glass membrane.

### 2.2 Surface Wettability

The wettability characteristics of the glass membranes were evaluated using contact angle measurements at different modification stages. Untreated glass membrane exhibits an intrinsic wettability due to the presence of many hydroxyl groups on the surface, which makes it hydrophilic. In air, the *θ*_W_ and *θ*_O_ of the untreated G2 glass membrane were ≈0°, and the *θ*_OW_ were 146.6 ± 2.1° (Figure S7, Supporting Information). Upon sulfobetaine modification, the *θ*_W_ and *θ*_O_ of G2‐SNF‐SB were ≈0° in air, and the *θ*_OW_ was 168 ± 2.5° (Figure 1D). The underwater oil droplet (1,2‐dichloroethane) on the sulfobetaine‐modified G2 glass membrane surface exhibits a super‐repellent spherical shape, as shown in Figure 1E. In addition, the *θ*_OW_ of other extracting agents was also tested, and the *θ*_OW_ of the extracting agent of sulfobetaine‐modified G2 glass membrane was greater than 160° (Figure 1F). The results indicated that the sulfobetaine‐modified glass membrane shows excellent underwater superoleophobicity.

### 2.3 Anti‐Fouling Performance

The fouling‐resistant ability of sulfobetaine‐modified glass membrane was investigated by underwater dynamic oil adhesion experiments. Figure 1G,H shows an oil droplet successively contact, press, and detach from the surface of the glass membrane under water after being released from a needle. For the unmodified glass membrane, the oil droplets eventually adhered to the membrane surface, undergoing such procedures (Figure 1G). However, when the sulfobetaine‐modified glass membrane undergoes the same process, the oil droplet could be easily detached from the membrane surface without any residue (Figure 1H). To further evaluate the membrane's excellent oil repellency, a 10 uL size drop of oil (1,2‐dichloroethane) was placed on the membrane surface, and when the sulfobetaine‐modified membrane surface was tilted to 2.6°, the oil droplet started to roll downwards without any stagnation (Figure S8A, Supporting Information), which indicated that the membrane was sufficiently oleophobic and the oil adhesion was extremely low.

To prevent the accumulation of oil, the membrane surface was required to repel oil droplets quickly upon contact. To simulate this process, a bunch of light oil (petroleum ether dyed with oil red O) was fast jetted on the surface of the glass membrane underwater. It was found that when petroleum ether was rapidly injected onto the surface of sulfobetaine‐modified glass membrane, the oil immediately all bounced off from the surface without leaving any traces, indicating that the sulfobetaine‐modified membrane could effectively resist the continuous impact of the oil droplets and prevent the formation of the oil cake layer. (Figure S8B, Supporting Information). To further test the anti‐oil‐fouling properties of the glass membrane, a drop of crude oil was dropped on the surface of the pre‐wetted glass membrane. As water continues to be injected, it can be clearly seen that crude oil spontaneously floats off the sulfobetaine‐modified glass membrane (Figure S8C, Supporting Information). These results demonstrated that the sulfobetaine‐modified glass can effectively resist the adhesion and accumulation of contaminants, showcasing exceptional self‐cleaning performance and oil resistance properties.

### 2.4 Extraction and Separation of Organic Pollutants

**Figure 2:**
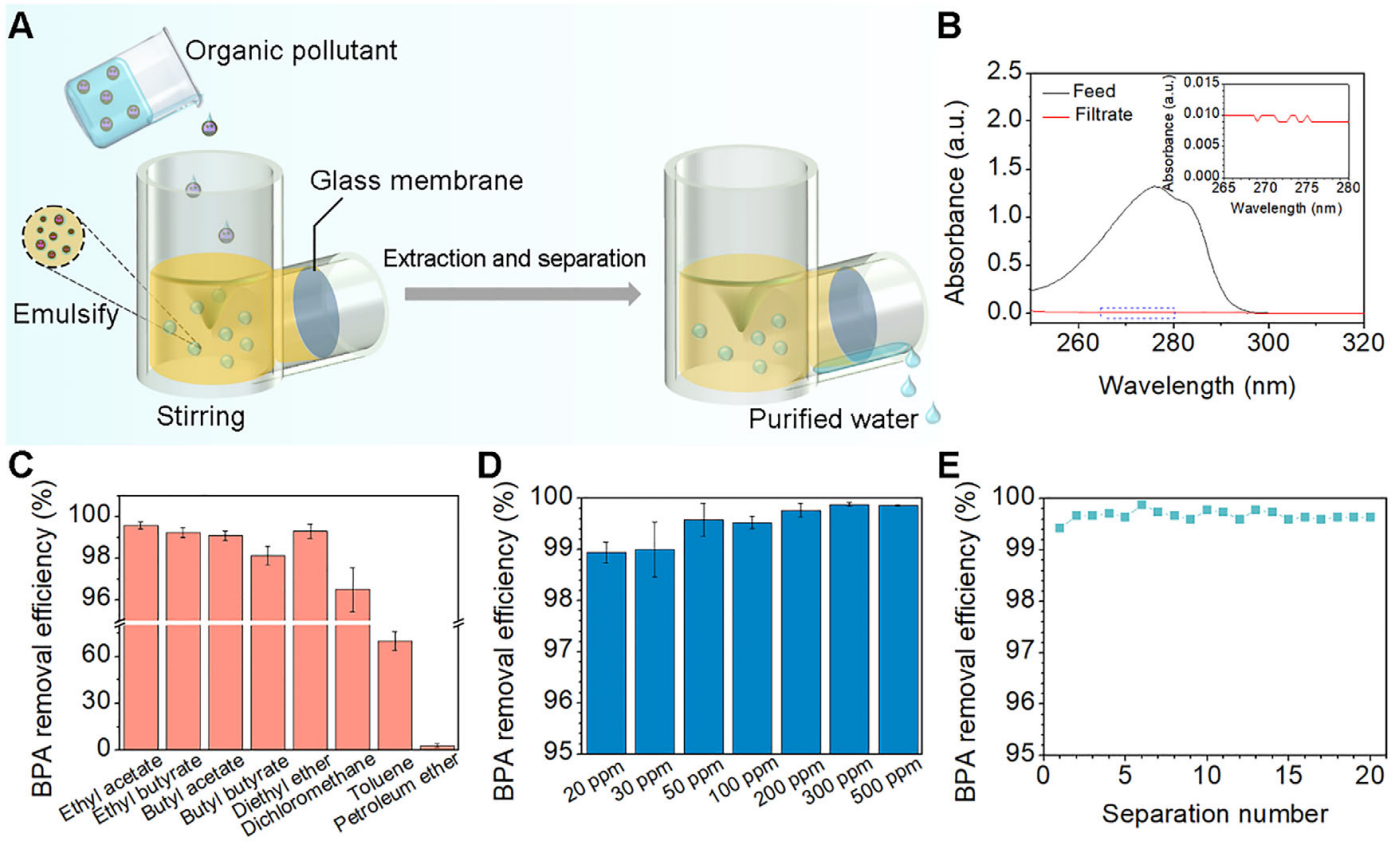
Extraction and separation of BPA. A) Schematic diagram of the extraction and separation of BPA. B) UV–vis spectra of BPA before and after extraction and separation. C) Removal efficiency of 100 ppm BPA with different extractants. D) Removal of different concentrations of BPA. E) Removal efficiency of BPA for 20 times extraction and separation for 100 ppm BPA.

We homemade a continuous in situ extraction and separation unit by assembling the SHUSO membrane filter on the side of an extractor. The extractor is responsible for the removal of organic contaminants from the water, and the filter on the side is used for the separation of the water phase and oil phases (**Figure** **2**A). Firstly, the glass filter was pre‐wetted with water. After that, the extractant was added, and the organic pollutant was poured into the extraction and separation unit under vigorous stirring, during which the glass filter automatically separates the extractant from the water. Given that bisphenol A (BPA) is a suspected endocrine disruptor and widely found in industrial wastewater, potentially leading to reproductive abnormalities, neurodevelopmental abnormalities, and even cancer,^[^ ^48^ ^]^ we chose BPA as a model organic pollutant to investigate the extraction and separation performance of this system. Here, ethyl acetate was selected as the extractant, and an aqueous solution containing 100 ppm of BPA was used as the wastewater sample. After sufficient mixing with the extractant, the water phase subsequently flowed out and was collected, while the extractant remains in the separation unit due to different wettability (Figure 2A; Figure S9, Supporting Information). The UV–vis spectra showed a strong BPA absorption peak at 276 nm in the feed solution, which was barely detectable after extractant enrichment (Figure 2B). The removal efficiency of BPA can reach 99.5% according to the standard curve (Figure S10, Supporting Information). This indicates that superwettable‐membrane‐assisted extraction and separation were capable of effectively removing organic pollutants from water.

Subsequently, the extraction and separation performance of different extractants for BPA was tested. As shown in Figure 2C, ester (ethyl acetate, butyl acetate, ethyl butyrate) and ether achieved more than 98.1% removal efficiency of BPA, while petroleum ether had only 3% removal efficiency. We believe that such a difference in removal efficiency results from different polarities of extractants: different from petroleum ether with low polarity, esters and ethers have higher polarities to form hydrogen bonds with the hydroxyl group of BPA and show a higher extraction efficiency. Actually, evidence of hydrogen bonds formation can be obtained by Fourier Transform Infrared Spectroscopy (FTIR), as displayed in Figure S11 (Supporting Information). The stretching vibration peak of the C═O bond for pure ethyl acetate appears at 1769 cm^−1^; however, upon increasing the concentration of BPA in ethyl acetate, a new peak at 1708 cm^−1^ was observed which can be assigned to the vibration peak of the C═O bond interacting with the hydroxyl group of BPA, implying the hydrogen bonds formation between ethyl acetate and BPA molecules.^[^ ^49^ ^]^ The partition coefficients of BPA in different extractants were also determined (Table S1, Supporting Information). The partition coefficient values followed the order: ethyl acetate (480.9±82.6) > diethyl ether (203.2±16) > ethyl butyrate (138±14.6), butyl acetate (112.8±25.1), butyl butyrate (76.4±10.3), and > dichloromethane (36.1±2.6) > toluene (7.7±2.5) > petroleum ether (0.6±0.1), which is in perfect agreement with the removal efficiency trend showing in Figure 2C.

Subsequently, ethyl butyrate was chosen to longitudinally investigate the extraction and separation performance for organic pollutants. For BPA in the concentration range of 20−500 ppm, the extraction and separation efficiencies for BPA were between 98.9% and 99.9% (Figure 2D). In addition, the durability of the extraction and separation was evaluated by repeating the extraction and separation of 100 ppm BPA over 20 times; and 100 mL of water filtrate was collected each time. Remarkably, the removal of BPA remained stable over 20 times of continuous extraction and separation, and was able to maintain a removal efficiency higher than 99.4% during the extraction and separation process (Figure 2E). To investigate the stability of the surface‐modified membrane, SEM observations, XPS measurements, and surface wettability analysis after extraction and separation of 100 ppm BPA for 20 times were performed. In all cases, there is no detectable change between the results after using 20 times and that of the freshly prepared samples (Figure S12, Supporting Information), indicating that the sulfobetaine‐modified glass membranes exhibit excellent stability. In addition, when the sulfobetaine‐modified glass membrane was pre‐wetted with water, oil could not penetrate through the modified membrane after continuous stirring for 6 h (Figure S13, Supporting Information), which was attributed to the fact that the sulfobetaine‐modified glass membrane could form a stable water‐binding layer with water and prevent the membrane surface from being contaminated by oil. Therefore, it enables the extraction and separation unit to ensure safe operation of continuous extraction and separation for long periods of time.

**Figure 3:**
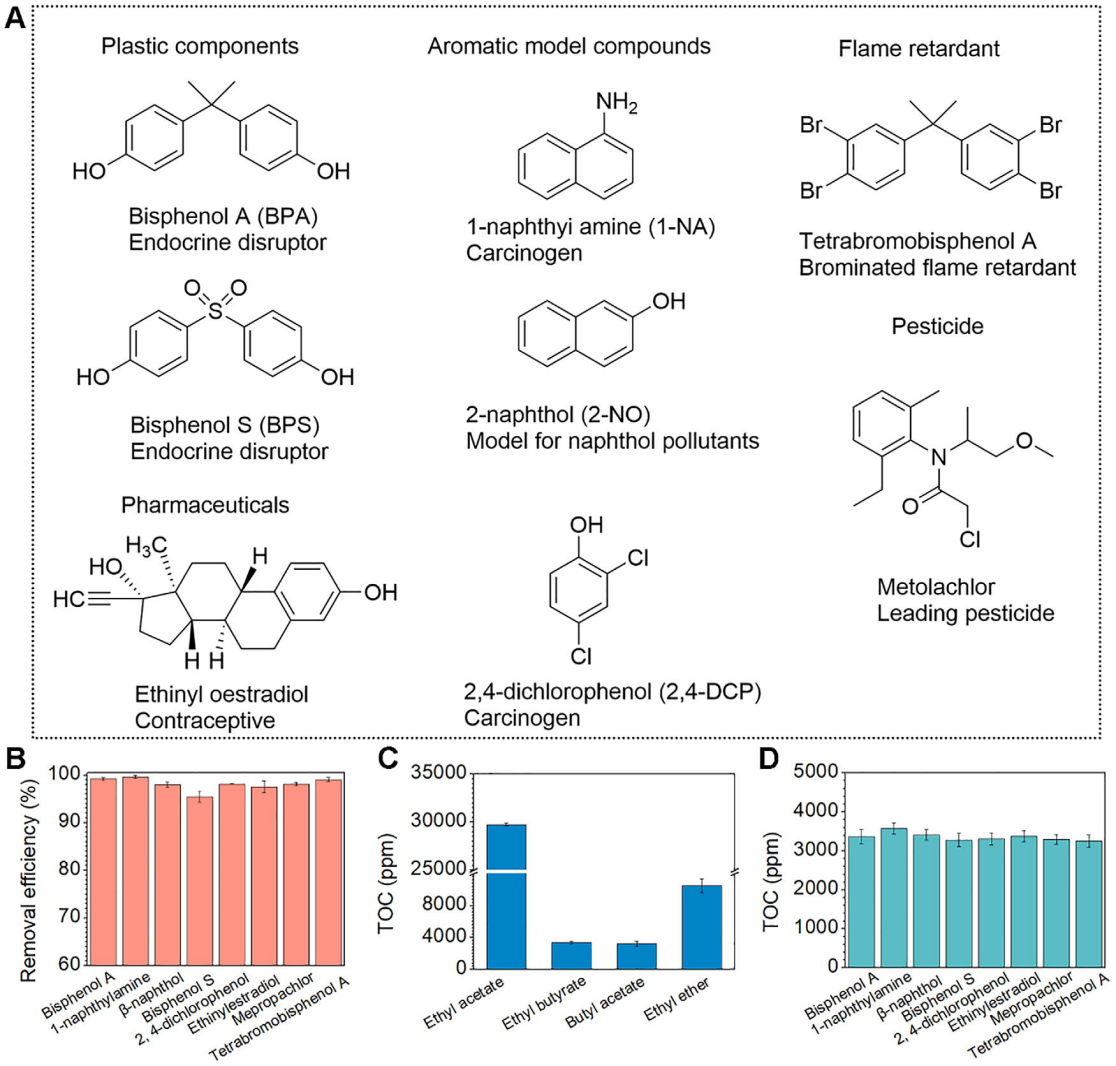
Various organic pollutants are removed by extraction and separation. A) Structures of each tested organic pollutant. B) Removal performance for different organic pollutants from a water sample, ethyl butyrate as extractant. C) TOC of filtrates after extraction and separation of 100 ppm BPA with different extractants. D) TOC of filtrates after extraction and separation of different organic pollutants, ethyl butyrate was used as an extractant.

In addition to BPA, we also evaluated the extraction and separation performance for the plastic components, pharmaceutical, pesticide, aromatic model compounds, and flame retardant, are shown in **Figure** **3**A. Extraction and separation performance studies of each of these compounds were performed similarly to those for BPA (100 ppm), except for ethinyl oestradiol (50 ppm), which was tested at a lower concentration because of its low water solubility. As shown in Figure 3B, each pollutant was efficiently removed from the water with an efficiency above 95.5%. These results indicate that extraction and separation based on superwettability can effectively remove a variety of highly concentrated organic pollutants and has great potential for application in practical wastewater treatment processes.

Liquid‐liquid extraction is the use of substances in different components assigned to two immiscible solvents in different solubility or distribution ratios to achieve the purpose of purification.^[^ ^50^ ^]^ After the extraction of the target compounds by a specific solvent, most of the target compounds can be transferred to the solvent phase, while the raffinate will contain a certain concentration of extractant. Thus, although organic pollutants from water can be effectively removed through extraction possess, a large amount of extractant remains in the filtrated water, resulting in the reintroduction of new pollutants into the water. As shown in Figure 3C, when ethyl acetate was used as the extractant, the TOC content of the extractant remaining in the filtrated water can reach 30 000 ppm after extraction and separation of BPA. Other extractant TOCs, such as ethyl butyrate, butyl acetate, and ether residues in filtered water, are ≈3400, 3300, and 11 000 ppm, respectively. When using ethyl butyrate as an extractant, the average TOC value of the collected water after extraction and separation of various organic pollutants was up to 3400 ppm. (Figure 3D). As a result, while efficient removal of organic pollutants was achieved, new pollutants are reintroduced into the water.

### 2.5 Multi‐Stage Extraction and Separation of Organic Pollutants

**Figure 4:**
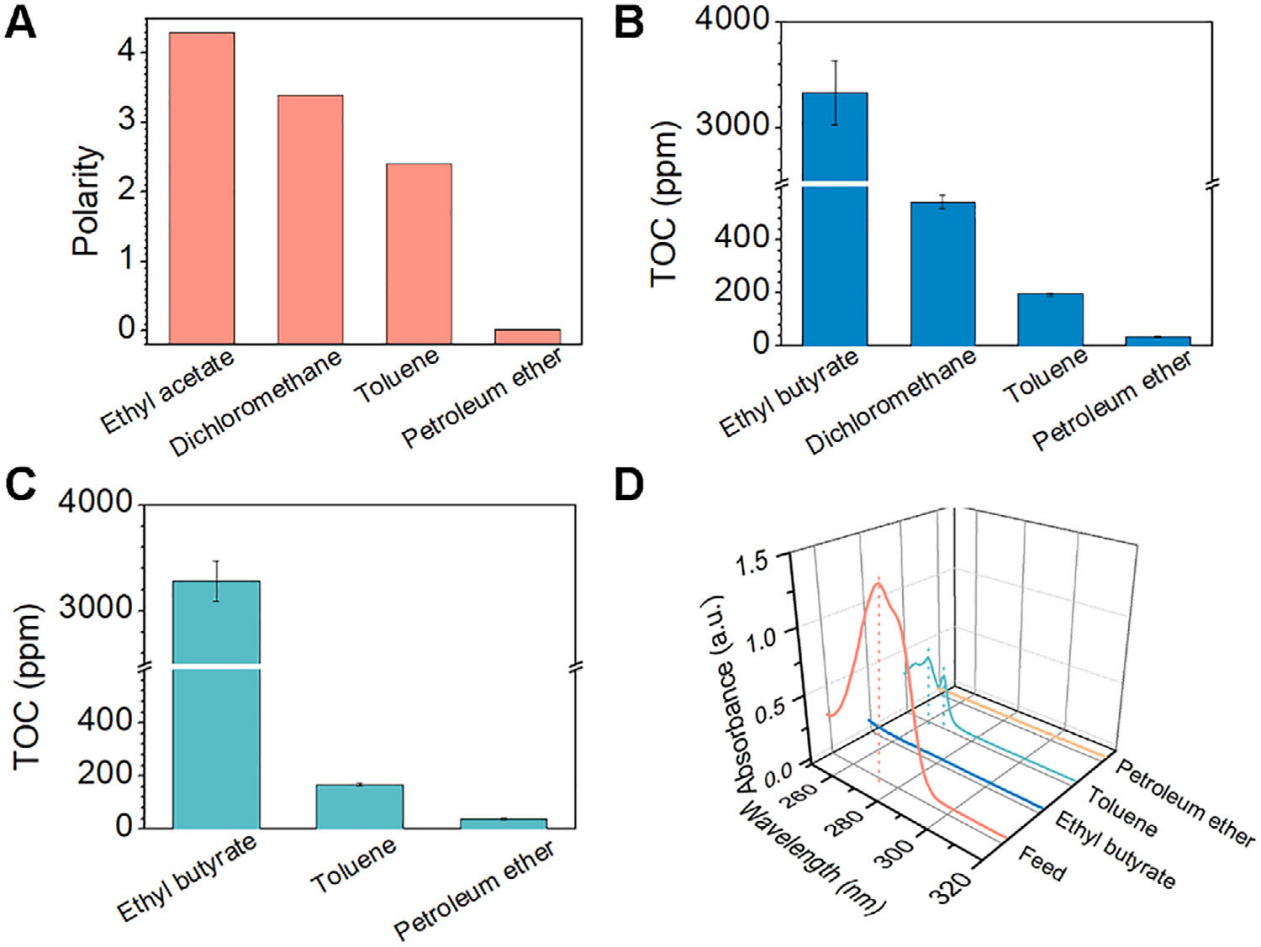
Multi‐stage extraction and separation of organic pollutants. A) Polarity of the selected extractant. B) TOC values of filtrate at each stage after multi‐stage extraction and separation of 100 ppm BPA. C) TOC values of filtrate at each stage after multi‐stage extraction and separation of 100 ppm BPA, ethyl butyrate was used as an extractant. D) UV–vis spectra of filtrate at various stages after multi‐stage extraction and separation of 100 ppm BPA.

In order to solve the above issues, we selected extractants such as ethyl acetate, dichloromethane, toluene, and petroleum ether based on the principle of similar solubility (**Figure** **4**A) and created a multi‐stage extraction and separation system to eliminate the residual extractants in the water. To verify the feasibility of our approach, first, ethyl acetate‐water mixtures were used to verify the ability of extraction and separation to eliminate raffinate. After the ethyl acetate‐water mixture was separated by multi‐stage extraction and separation system, from an initial TOC value of 30 000 ppm in the filtered water, the TOC content in the collected water was reduced to 740 ppm after the purification at the dichloromethane stage, indicating that dichloromethane was able to effectively extract the high residual ethyl acetate in the water (Figure S14, Supporting Information). This inspired us to further use organic solvents of similar polarity, such as toluene and petroleum ether for the extraction and separation of the residual high concentration of extractant. After the above filtered water was extracted and separated by a stage of toluene, the TOC value was reduced to 270 ppm, and after the final stage of petroleum ether, the TOC value was surprisingly reduced to 48 ppm. Similarly, when the ethyl butyrate‐water mixture was subjected to multi‐stage purification, the final collected water had a TOC value as low as 38 ppm (Figure S15, Supporting Information). The results show that the superwettable‐membrane‐assisted multi‐stage extraction and separation system was an effective way to eliminate the high concentration of extracts in water.

Subsequently, 100 ppm BPA was used to verify the separation ability of the multi‐stage extraction and separation system. After separation in a multi‐stage extraction and separation system consisting of ethyl butyrate, dichloromethane, toluene, and petroleum ether, the TOC values in the collected water were reduced from 3300 to 540 ppm, then to 194 ppm, and finally to 36 ppm (Figure 4B). To improve the cost‐effectiveness and simplicity of the multi‐stage extraction and separation system, we first attempted to eliminate the extraction and separation step involving dichloromethane. After separation of 100 ppm BPA by a multi‐stage extraction and separation system, the final collected water had a TOC value of 38 ppm (Figure 4C). From the UV results, it was observed that the absorption peak of BPA was hardly observed in the final collected water (Figure 4D). In addition, we attempted to further subtract the toluene stage, and the final filtrate obtained after extraction and separation had a TOC value of 75 ppm (Figure S16, Supporting Information), probably due to the difficulty of petroleum ether alone to completely and effectively extract the high residual ethyl butyrate in the water. Based on the results obtained above, a multistage extraction and separation system based on ethyl butyrate, toluene, and petroleum ether was used for the purification of organic pollutants.

**Figure 5:**
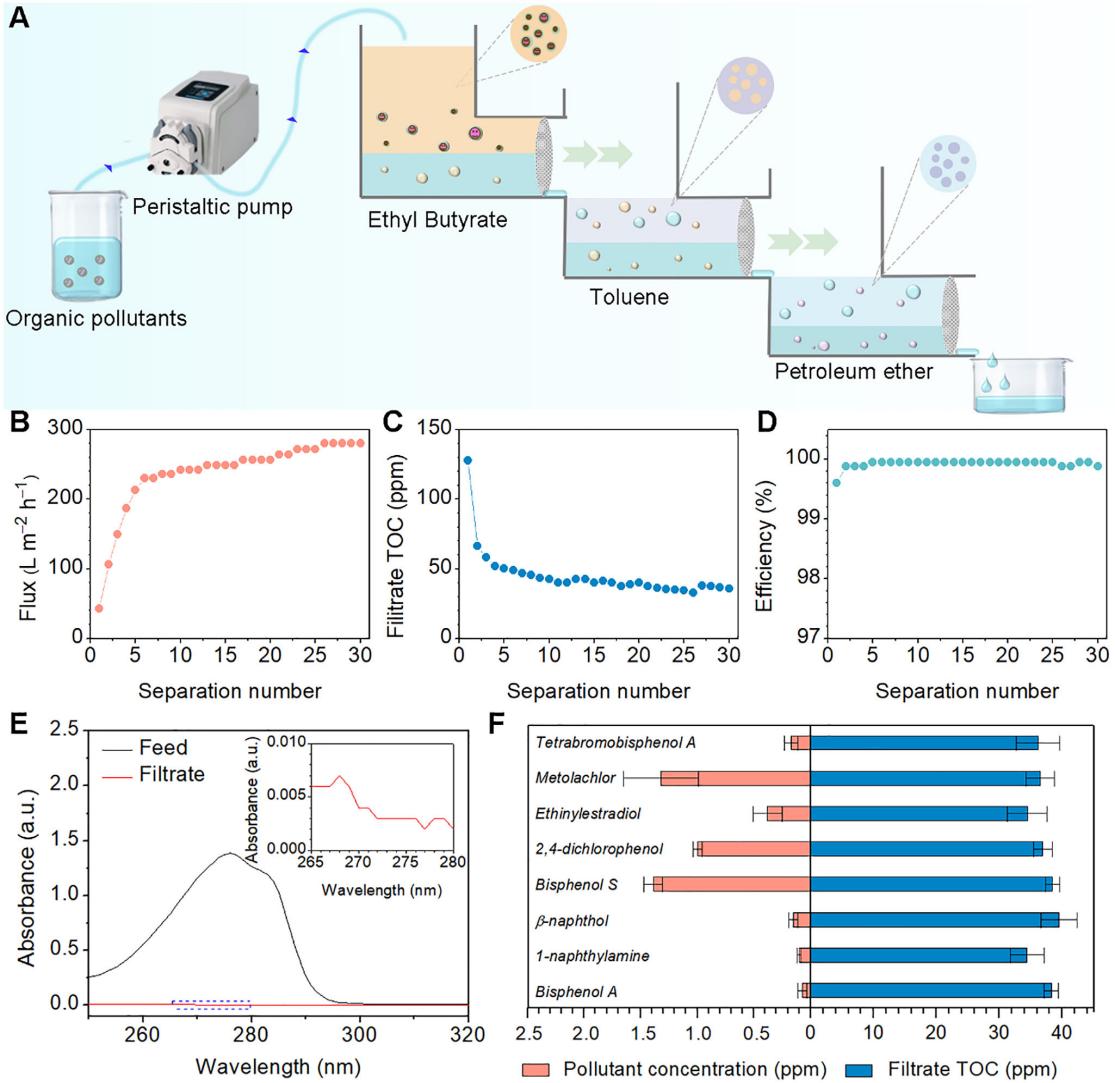
Simultaneous extraction and separation of organic pollutants and residual extractants. A) Schematic diagram of a multi‐stage extraction and separation system connected in series. B) Flux for the separation of 100 ppm BPA during 30 times continuous extraction and separation. C) TOC for the separation of 100 ppm BPA during 30 times continuous extraction and separation. D) Removal efficiency for the separation of 100 ppm BPA during 30 times of continuous extraction and separation. E) UV–vis spectra of 100 ppm BPA before and after multi‐stage extraction and separation. F) TOC and residual pollutant content of filtrates after extraction and separation of different organic pollutants.

To make multi‐stage extraction and separation systems more practical and convenient, we further designed a multi‐stage extraction and separation system by connecting the extraction and separation units in series (**Figure** **5**A; Figure S17, Supporting Information). Given the oil‐water mixture will form a surfactant‐free oil‐water emulsion under continuous mixing and G3 filters are capable of efficiently separating surfactant‐free oil‐water emulsions,^[^ ^51^ ^]^ subsequent separation tests were performed using G3 glass filter in order to ensure efficient separation. Subsequently, we explored the separation performance of a multi‐stage extraction and separation system, where each 20 mL of collected water was used as a cycle, the flow rate of the peristaltic pump was 5 mL min^−1^.

In the process of continuous separation, the separation flux gradually became larger with the increase of the number of separations, with the 10^th^ separation having a flux of 242 L m^−2^ h^−1^ and the 30^th^ separation having a flux of 280 L m^−2^ h^−1^ (Figure 5B). The TOC values in the collected water filtrate also gradually decreased, with the 10^th^ separation having a TOC value of 42 ppm, and the 30^th^ separation having a TOC value of 36 ppm (Figure 5C). In addition, the removal efficiency of the BPA was maintained at almost 100% (Figure 5D). The results of UV–vis showed that the absorption peaks of BPA were hardly observed in the collected water after purification by a multi‐stage separation and extraction system. (Figure 5E). Based on the above separation model, the separation performance of a wide range of organic pollutants was evaluated, and it can be obtained that the TOC of the collected water was ≈40 ppm in all cases, and the average content of pollutants in the collected water filtrate was less than 1.5 ppm (Figure 5F). It is worth noting that the above process successfully combines extraction and separation with the great advantage of simplicity and high efficiency, which will make extraction and separation more practical in water treatment.

We also investigated the removal efficiency of BPA as a function of peristaltic pump flow rate (5, 10, and 15 mL min^−1^) for the multi‐stage extraction and separation system. As exhibited in Figure S18 (Supporting Information), a slight difference in removal efficiency was observed for the first three cycles; however, the removal efficiency maintains almost a constant value close to 100% for the rest cycles, regardless of the flow rate.

## 3 Discussion

### 3.1 Synergistic Effect of the Combined Individual Extraction and Membrane Processes

Highly efficient removal of organic pollutants from water is crucial for environmental sustainability.^[^ ^1^ ^]^ To demonstrate the advantages of our novel superwettable‐membrane‐assisted extraction and separation technology, we conducted a comparative analysis for separating 100 ppm BPA using the individual liquid‐liquid extraction, the individual membrane separation, and our novel method (Table S2, Supporting Information). Although the individual liquid‐liquid extraction can remove BPA from water efficiently with a removal efficiency 99.28%, the TOC value of the collected filtrate increased remarkably to 3400 ppm, i.e., ≈44 times higher than that of the original BPA water solution. This is due to the fact that the extractant of ethyl butyrate was solubilized in the water phase during the liquid‐liquid extraction process. Individual membrane separation (i.e., direct membrane filtration) with a G2 glass filter after surface modification was incapable of removing BPA from water. The removal efficiency and TOC of the collected filtrate are 0% and 78 ppm, respectively. This is because the pore size of the glass filter (30–50 µm) is considerably larger than the dimensions of the BPA molecules (less than 1–2 nm), resulting in the failure of the selective sieving and size exclusion that is key to membrane filtration. On the contrary, our novel superwettable‐membrane‐assisted extraction and separation technology can separate BPA from water more efficiently with a removal efficiency of 99.93%, indicating that hardly any BPA residue remains in the water filtrate. Moreover, the TOC value of the collected water filtrate was as low as 38 ppm. Obviously, our novel technology effectively resolves the conflict between high separation efficiency and secondary pollution, providing a more sustainable solution for organic pollutant removal.

### 3.2 Mechanism of the Superwettable‐Membrane‐Assisted Extraction and Separation

It is worth mention that the underlying separation mechanism of our novel technology for removing organic pollutants is related to two main issues, the liquid‐liquid extraction (Key Step I) and superwettable oil‐water separation (Key Step II), both of which are highly correlated with interfacial behavior. In Key Step I, the organic pollutants in the wastewater are efficiently transferred from the water phase to the immiscible extractant phase through strong liquid‐liquid interface interaction following the Nernst Distribution Law, in which the organic pollutants’ partition coefficient in the extractant phase and the water phase plays an important role. In fact, it was clearly found that the removal efficiency of the organic pollutants in this single step mainly depends on the nature of the extractant. The higher the extractant's partition coefficient, the greater the removal efficiency, following the order: ethyl acetate > diethyl ether > ethyl butyrate > butyl acetate > butyl butyrate > dichloromethane > toluene > petroleum ether (Table S1, Supporting Information). After the Key Step I, almost all of the organic pollutants are removed from the water phase; however, the extractant can possibly enter the water phase, the mount of which depends mainly on the polarity of the extractant. Next, in Key Step II, the new contaminant of the extractant that was used in Key Step I must be removed with high efficiency, which was carried out based on the principle of superwettable oil‐water separation, involving strong interfacial interactions between the extractant phase, the water phase, and the porous solid phase. Due to the superhydrophilicity and simultaneous underwater superoleophobicity, the sulfobetaine‐modified porous glass filter membrane allows only water to pass through. The remaining oil residue (i.e., the residual extractant) in the final water filtrate is largely affected by the oil‐water separation efficiency for different oil extractants. For example, the oil‐water separation efficiency of petroleum ether (>99.995%) is greater than ethyl butyrate (99.7%), resulting in a lower TOC value in the final water filtrate for petroleum ether as compared to ethyl butyrate. From the perspective of contaminant residue in the filtrate, petroleum ether is a superior solvent to ethyl butyrate. However, in Key Step I, ethyl butyrate exhibits greater extraction capacity for organic pollutants than petroleum ether. Taking into account both of the above Key Steps, the selection principle for the extractant should balance its extraction capacity for organic pollutants with its own residual levels in the final water filtrate. Consequently, guiding by the above separation mechanism, an optimal extractant selection strategy was final proposed: ethyl butyrate was used as the first extractant to remove the organic pollutants through superwettable‐membrane‐assisted extraction and separation, and subsequently other solvents such as toluene and/or petroleum ether are used to remove the first extractant; finally, the organic pollutants in the original wastewater are removed thoroughly from the water, accompanied with a very low TOC value.

### 3.3 Comparison Between Conventional Membrane Filtration and Superwettable‐Membrane‐Assisted Extraction and Separation

Certainly, it is possible to remove organic pollutants such as BPA by direct membrane filtration if the pore size of the membrane is small enough, relying on the selective sieving and size exclusion effect. However, it still face the following problems:^[^ ^52^ ^]^ 1) the pore size of the membrane must be between that of the molecular sizes of the organic pollutants and the solvents (smaller than the sizes of the solvents molecules but bigger than that of solvent molecules, usually less than a few nanometers), making the design and regulation of the membrane pores very challenging;^[^ ^14^ ^]^ 2) high transmembrane pressures ranging from 2 to 15 bar are normally required to achieve efficient removal of organic pollutants from water due to the extremely small sizes of the membranes, resulting in high energy consumption;^[^ ^53^, ^54^ ^]^ 3) the permeability of the membrane is very low, often in the range of 5−50 L m^−2^ h^−1^ bar^−1^.^[^ ^15^ ^]^ Compared to membrane separation based on selective sieving and size exclusion, the hybrid system created in this work through the integration of liquid‐liquid extraction and in situ superwettable membrane separation show many advantages: 1) the pore size of the membrane is significantly larger (30−50 µm) and precise control of the pore size is not required; 2) the transmembrane pressure, provided by the gravity of the liquid above the membrane is low (≈10^−2^ bar), which means that the separation process is energy efficient; 3) the separation flux is as high as 280 L m^−2^ h^−1^, which corresponds to a permeability of 28 000 L m^−2^ h^−1^ bar^−1^, hundreds times higher than the permeability of membrane separation based on selective sieving and size exclusion.

### 3.4 Simultaneous Removal of Multiple‐Component Pollutants

Most of the wastewater caused by daily activities and various industrial processes constitutes a complex system which typically involve not only single types of pollutants but also multiple‐component pollutants in mixed form.^[^ ^52^ ^]^ Consequently, a multiple‐component pollutant mixture containing 100 ppm of BPA, 1‐naphthylamine, tetrabromobisphenol A, ethinyl oestradiol, and metolachlor was prepared and was used as a model complex wastewater system so as to demonstrate the great potential of the superwettable‐membrane‐assisted extraction and separation technology in the treatment of complex wastewater systems. The UV–vis spectra indicated that organic pollutants in the feed solution exhibited a strong absorption peak at 250–320 nm, which was barely detectable after multi‐stage extraction and separation (Figures S19 and S20, Supporting Information). The TOC value in the final collected water filtrate was as low as ≈33 ppm. The results demonstrate that the system exhibits robust performance and high removal efficiency when treating complex multi‐component wastewater, indicating its significant potential for practical industrial applications.

## 4 Conclusion

In summary, liquid‐liquid extraction for the treatment of organic pollutants is simple, low energy consumption and high efficiency, but requires tedious treatment steps, and the residual extracts need to be further purified to prevent secondary contamination. Membranes with special wettability offer an ideal method for highly efficient removal of oil from water. Herein, a novel strategy combining liquid‐liquid extraction with superwettable membrane separation was applied to remove organic pollutants. In order to improve the separation performance and resistance to oil contamination of glass membranes, the membranes were modified by sulfobetaine molecules with strong resistance to oil contamination through click chemistry. The results showed that the sulfobetaine‐modified membranes had excellent self‐cleaning properties and resistance to oil contamination. Subsequently, we fabricated a homemade device for continuous in situ extraction and separation by assembling the sulfobetaine‐modified porous glass membranes on the side of glass containers. The superhydrophilic‐underwater superoleophobic properties of the sulfobetaine‐modified glass filters enable the water phase to be automatically and rapidly separated from water samples mixed with extractants and organic pollutants. The research results showed that the synergistic integration of extraction and superwettable membrane separation technologies enables a continuous and efficient elimination of plastic constituents, pharmaceuticals, pesticides, and aromatic model compounds from water bodies. The extraction and separation efficiency of various pollutants in water can reach above 95.5%. However, while effectively removing organic pollutants, a large amount of residual extractant can remain in the water, leading to the generation of new pollutants. Subsequently, based on the theory of similarity and intermiscibility, we have designed a multi‐stage extraction and separation system by connecting the multistage of extraction and separator together in series. After multiple stages of extraction and separation, the final TOC value in the collected water was less than 40 ppm, alongside a residual pollutant concentration of under 1.5 ppm. As a result, the superwettable‐membrane‐assisted extraction and separation system demonstrated a great potential for application in the field of wastewater treatment.

## 5 Experimental Section

### Materials and Chemicals

Ethyltrichlorosilane (ETCS), (3‐Mercaptopropyl)triethoxysilane, 1,3‐propanesulfonate, *N,N*‐Dimethylallylamine, 2,2‐bimethoxy‐2‐phenylacetophenone (DMPA), bisphenol A (BPA), 1‐naphthylamine, β‐naphthol, bisphenol S, 2,4‐dichlorophenol, ethinylestradiol, tetrabromobisphenol A, oil red O, and sodium hydroxide (NaOH) were obtained from Macklin Chemical Reagent Co., Ltd. (Shanghai, China). Metolachlor was purchased from Sigma–Aldrich Chemical Reagent Co., Ltd. Crude oil was obtained from China Petroleum & Chemical Corporation (SINOPEC) Shengli Oilfield. Water samples of organic pollutants were prepared using deionized water. Pollutant model compounds were obtained from commercial sources. Porous glass filter Grade G2 (pore size = 30–50 µm) and G3 (16–30 µm) were commercial products.

### Characterizations

The surface morphology was performed on a JSM‐7610FPlus Field‐emission Scanning Electron Microscopy (JEOL, Japan). The viscosity of the crude oil was determined by a MCR 302e Rheometer (Anton Paar, Austria). The chemical composition of unmodified and modified glass membranes was obtained by the Escalab 250Xi X‐ray photoelectron spectroscopy (Thermo Fisher, USA). The water contact angle in air (*θ*_W_), oil rolling angle underwater, oil contact angle in air (*θ*_O_), and oil contact angle underwater (*θ*_OW_) were measured by an LSA100 contact angle measurement instrument (LAUDA Scientific, Germany). The oil content in the filtrates was determined by a TOC‐L/CPN total organic carbon analyzer (TOC, Shimadzu, Japan). The thiol‐ene click reaction was triggered using an EP‐U4545K‐A3, UV lamp (365 nm, Epileds, China). UV–vis spectroscopy was performed on a UV‐1900i UV–vis spectrometer (Shimadzu, Japan). FTIR spectra were performed on a Nicolet iS20 spectrometer (Thermo‐Fisher Scientific, USA).

### Surface Functionalization of Porous Glass Membrane

Here, the preparation of sulfobetaine‐modified G2 membrane was an example, and the preparation of sulfobetaine‐modified G3 membrane was the same as the following steps. Firstly, silicone nanofilament‐coated porous glass membranes were fabricated via chemical vapor deposition using ETCS as a precursor.^[^ ^51^ ^]^

Sulfobetaine‐modified glass membranes were prepared via thiol‐ene click chemistry. Firstly, the SNF‐modified glass membrane was heated to 450 °C to obtain the SNF‐OH‐modified glass membrane. Subsequently, SNF‐OH‐modified glass membrane was added to 150 mL of toluene solution, and bubbled with nitrogen for 30 min. Then, (3‐mercaptopropyl)triethoxysilane (1.5 mL) was added and heated at 110 °C for 1 h to obtain thiol‐functionalized glass membranes.

Synthesis of 3‐(allyldimethylammonio)propane‐1‐sulfonate. *N,N‐*dimethylallylamine (20 mmol, 1.703 g) and 1,3‐propanesulfonic acid (21 mmol, 2.57 g) were added to acetone (40 mL) and then heated to reflux for 8 h. After cooling to room temperature, the solvent was removed by filtration, and the product was washed three times with acetone.

The thiol‐functionalized glass membrane was immersed in 20 mL of methanol solution containing 3‐(allyldimethylammonio)propane‐1‐sulfonate (0.1 g) and DMPA (4.1 mg). Then, the solution was irradiated under a 365 nm UV light for 5 min at room temperature, cleaned with methanol, and dried in an oven to afford the sulfobetaine‐modified glass membrane.

### Extraction and Separation of Organic Micropollutants

The purification performance of extraction and separation for organic pollutants was investigated in aqueous solutions with a feed concentration of 100 ppm, with the exception of ethinyl estradiol (50 ppm), which was tested at a lower concentration due to its low aqueous solubility. All studies were conducted at ambient temperature on a stirring, and the procedure was identical for all the organic pollutants tested. The extraction and separation unit was assembled by combining a glass filter and a homemade glass bottle with parallel outlets, and the glass membrane needs to be prewetted with water to the extraction and separation tests.

To test the removal efficiency of organic pollutants, 100 mL of extractant was added to the extraction and separation unit, and then 100 mL of the water sample was added to the extractor under continuous stirring. In order to remove the residual extracts, a multi‐stage extraction and separation unit was designed by connecting several separation units in series. The residual concentration of the organic pollutant in the water was detected by UV–vis, and the organic carbon content in the water was analyzed by TOC. The separation and extraction efficiency of the organic pollutant was calculated by Equation (1):

(1) Efficiency=(1−C/C0)×100%

where C_0_ = initial organic pollutant concentration, C = concentration of pollutants in filtrate.

## Conflict of Interest

The authors declare no conflict of interest.

## Supporting information



Supporting Information

## Data Availability

The data that support the findings of this study are available from the corresponding author upon reasonable request.

## References

[1] A. Alsbaiee , B. J. Smith , L. Xiao , Y. Ling , D. E. Helbling , W. R. Dichtel , Nature 2015, 529, 190. 26689365 10.1038/nature16185

[2] T. Brodin , J. Fick , M. Jonsson , J. Klaminder , Science 2013, 339, 814. 23413353 10.1126/science.1226850

[3] S. D. Richardson , T. A. Ternes , Anal. Chem. 2014, 86, 2813. 24502364 10.1021/ac500508t

[4] M. Patel , R. Kumar , K. Kishor , T. Mlsna , C. U. Pittman , D. Mohan , Chem. Rev. 2019, 119, 3510. 30830758 10.1021/acs.chemrev.8b00299

[5] D. Gokhale , A. F. Hamelberg , P. S. Doyle , Nat. Water 2024, 2, 62.

[6] C. Cheng , Y. Cai , G. Guan , L. Yeo , D. Wang , Angew. Chem., Int. Ed. 2018, 57, 11177. 10.1002/anie.201803834 29964347

[7] X. Hu , G. Xu , H. Zhang , M. Li , Y. Tu , X. Xie , Y. Zhu , L. Jiang , X. Zhu , X. Ji , Y. Li , A. Li , ACS Appl. Mater. Interfaces 2020, 12, 12165. 32057224 10.1021/acsami.0c00597

[8] H. Abedpour , J. S. Moghaddas , M. N. Borhani , T. N. Borhani , J. Water Process Eng. 2023, 53, 103676.

[9] A. Sobek , S. Abel , H. Sanei , S. Bonaglia , Z. Li , G. Horlitz , A. Rudra , K. Oguri , R. N. Glud , Nat. Commun. 2023, 14, 2012. 37037817 10.1038/s41467-023-37718-z PMC10086072

[10] Y.‐J. Zhang , G.‐X. Huang , L. R. Winter , J.‐J. Chen , L. Tian , S.‐C. Mei , Z. Zhang , F. Chen , Z.‐Y. Guo , R. Ji , Y.‐Z. You , W.‐W. Li , X.‐W. Liu , H.‐Q. Yu , M. Elimelech , Nat. Commun. 2022, 13, 3005. 35637224 10.1038/s41467-022-30560-9 PMC9151758

[11] L. Zhou , Z. Liu , Z. Guan , B. Tian , L. Wang , Y. Zhou , Y. Zhou , J. Lei , J. Zhang , Y. Liu , Appl. Catal., B 2020, 263, 118326.

[12] Y. Chen , X. Xie , X. Xin , Z.‐R. Tang , Y.‐J. Xu , ACS Nano 2018, 13, 295. 30507143 10.1021/acsnano.8b06136

[13] J. Zdarta , T. Jesionowski , M. Pinelo , A. S. Meyer , H. M. N. Iqbal , M. Bilal , L. N. Nguyen , L. D. Nghiem , Bioresour. Technol. 2022, 344, 126201. 34710611 10.1016/j.biortech.2021.126201

[14] N. A. Khan , S. Singh , E. A. López‐Maldonado , P. N. , P. F. Méndez‐Herrera , J. R. López‐López , U. Baig , P. C. Ramamurthy , N. M. Mubarak , R. R. Karri , I. H. Aljundi , Desalination 2023, 565, 116873.

[15] Y.‐L. Liu , X.‐M. Wang , H.‐W. Yang , Y. F. Xie , X. Huang , J. Membr. Sci. 2019, 572, 152.

[16] B. Zheng , X. Lin , X. Zhang , D. Wu , K. Matyjaszewski , Adv. Funct. Mater. 2019, 30, 1907006.

[17] L. Zhang , Y. Guo , R. Hao , Y. Shi , H. You , H. Nan , Y. Dai , D. Liu , D. Lei , J. Fang , Nat. Commun. 2021, 12, 6849. 34824226 10.1038/s41467-021-27100-2 PMC8617178

[18] S. R. Cotty , A. Faniyan , J. Elbert , X. Su , Nat. Chem. Eng. 2024, 1, 281.

[19] B. Hashemi , P. Zohrabi , K.‐H. Kim , M. Shamsipur , A. Deep , J. Hong , TrAC, Trends Anal. Chem. 2017, 97, 83.

[20] A. Bokhary , M. Leitch , B. Q. Liao , J. Water Process Eng. 2021, 40, 101762.

[21] S. F. Anis , R. Hashaikeh , N. Hilal , Desalination 2019, 468, 114077.

[22] B. Zhu , R. Shao , N. Li , C. Min , S. Liu , Z. Xu , X. Qian , L. Wang , Chem. Eng. J. 2022, 449, 137013.

[23] M. Zhao , X. Wang , S. Wang , W. Lu , M. He , M. Gao , Proc. Natl. Acad. Sci. USA 2025, 121, 2403072121.

[24] J. Mukherjee , B. K. Lodh , R. Sharma , N. Mahata , M. P. Shah , S. Mandal , S. Ghanta , B. Bhunia , Chemosphere 2023, 345, 140473. 37866496 10.1016/j.chemosphere.2023.140473

[25] D. Chen , Y. Cheng , N. Zhou , P. Chen , Y. Wang , K. Li , S. Huo , P. Cheng , P. Peng , R. Zhang , L. Wang , H. Liu , Y. Liu , R. Ruan , J. Cleaner Prod. 2020, 268, 121725.

[26] H. Li , X. Han , L. Zhang , W. Yu , W. Bie , M. Wei , Z. Wang , F. Kong , W. Wang , Carbohydr. Polym. 2023, 312, 120832. 37059548 10.1016/j.carbpol.2023.120832

[27] P. Wang , G. An , P. Jarvis , W. Liu , S. Ding , R. Qu , Z. Li , C. Ye , W. Chu , Chem. Eng. J. 2024, 482, 148826.

[28] Z. Xu , Z. Zhu , N. Li , Y. Tian , L. Jiang , ACS Nano 2018, 12, 10000. 30256616 10.1021/acsnano.8b04328

[29] A. Spietelun , Ł. Marcinkowski , M. de la Guardia , J. Namieśnik , Talanta 2014, 119, 34. 24401382 10.1016/j.talanta.2013.10.050

[30] Z. Chu , Y. Feng , S. Seeger , Angew. Chem., Int. Ed. 2014, 54, 2328. 10.1002/anie.201405785 25425089

[31] S. Gan , H. Li , X. Zhu , X. Liu , K. Wei , L. Zhu , B. Wei , X. Luo , J. Zhang , Q. Xue , Adv. Funct. Mater. 2023, 33, 2305975.

[32] J. Gong , B. Xiang , Y. Sun , J. Li , J. Mater. Chem. A 2023, 11, 25093.

[33] Y. Pang , Z. Yu , H. Chen , Q. Xiang , Q. Wang , C. Xie , Y. Liu , J. Hazard. Mater. 2022, 434, 128833. 35429755 10.1016/j.jhazmat.2022.128833

[34] D. Wang , H. Huang , F. Min , Y. Li , W. Zhou , Y. Gao , G. Xie , Z. Huang , Z. Dong , Z. Chu , Small 2024, 20, 2402946. 10.1002/smll.202402946 38881253

[35] H. Liu , Y. Wang , B. Zhu , H. Li , L. Liang , J. Li , D. Rao , Q. Yan , Y. Bai , C. Zhang , L. Dong , H. Meng , Y. Zhao , Adv. Mater. 2024, 36, 2311013. 10.1002/adma.202311013 38341656

[36] M. Chen , S. G. J. Heijman , M. W. J. Luiten‐Olieman , L. C. Rietveld , Water Res. 2022, 216, 118267. 35306459 10.1016/j.watres.2022.118267

[37] L. Yan , X. Yang , H. Zeng , Y. Zhao , Y. Li , X. He , J. Ma , L. Shao , J. Membr. Sci. 2023, 668, 121243.

[38] T. Wu , Y. Hou , Z. Liu , Y. Li , L. Wang , G. Wu , Z. Sheng , J. Sun , X. Zhang , Nat. Water 2024, 2, 899.

[39] D. Yadav , T. Chakraborty , A. Bedar , S. Saxena , S. Shukla , Sep. Purif. Technol. 2025, 367, 132851.

[40] H. Zhang , F. Wang , Z. Guo , Adv. Colloid Interface Sci. 2024, 325, 103097. 38330881 10.1016/j.cis.2024.103097

[41] A. M. C. Maan , A. H. Hofman , W. M. de Vos , M. Kamperman , Adv. Funct. Mater. 2020, 30, 2000936.

[42] Z. Xu , L. Li , J. Liu , C. Dai , W. Sun , J. Chen , Z. Zhu , M. Zhao , H. Zeng , J. Colloid Interface Sci. 2022, 608, 702. 34634545 10.1016/j.jcis.2021.09.123

[43] D. Wang , Y. Li , H. Huang , F. Min , W. Zhou , T. Zhang , Y. Gao , H. Liu , Z. Chu , Sep. Purif. Technol. 2024, 351, 128121.

[44] A. B. Asha , Y. Chen , R. Narain , Chem. Soc. Rev. 2021, 50, 11668. 34477190 10.1039/d1cs00658d

[45] Q. Li , C. Wen , J. Yang , X. Zhou , Y. Zhu , J. Zheng , G. Cheng , J. Bai , T. Xu , J. Ji , S. Jiang , L. Zhang , P. Zhang , Chem. Rev. 2022, 122, 17073. 36201481 10.1021/acs.chemrev.2c00344

[46] D. Dong , Y. Zhu , W. Fang , M. Ji , A. Wang , S. Gao , H. Lin , R. Huang , J. Jin , Adv. Funct. Mater. 2022, 32, 2113247.

[47] D. Wang , Y. Gao , S. Gao , H. Huang , F. Min , Y. Li , S. Seeger , J. Jin , Z. Chu , J. Membr. Sci. 2023, 670, 121336.

[48] L. Kan , W. Mu , C. Chang , F. Lian , Sep. Purif. Technol. 2023, 312, 123388.

[49] F.‐L. Luo , F.‐H. Luo , Q. Xing , X.‐Q. Zhang , H.‐Q. Jiao , M. Yao , C.‐T. Luo , D.‐J. Wang , Chin. J. Polym. Sci. 2013, 31, 1685.

[50] S. F. Hammad , I. A. Abdallah , A. Bedair , F. R. Mansour , J. Sep. Sci. 2021, 45, 185. 34472701 10.1002/jssc.202100452

[51] Z. Chu , S. Seeger , Adv. Mater. 2015, 27, 7775. 26501390 10.1002/adma.201503502

[52] N. K. Khanzada , M. U. Farid , J. A. Kharraz , J. Choi , C. Y. Tang , L. D. Nghiem , A. Jang , A. K. An , J. Membr. Sci. 2020, 598, 117672.

[53] H. Guo , Y. Deng , Z. Yao , Z. Yang , J. Wang , C. Lin , T. Zhang , B. Zhu , C. Y. Tang , Water Res. 2017, 121, 197. 28535433 10.1016/j.watres.2017.05.037

[54] Y. S. Khoo , P. S. Goh , W. J. Lau , A. F. Ismail , M. S. Abdullah , N. H. Mohd Ghazali , N. K. E. M. Yahaya , N. Hashim , A. R. Othman , A. Mohammed , N. D. A. P. Kerisnan , M. A. M. Yusoff , N. H. F. Hashim , J. Karim , N. S. Abdullah , Chemosphere 2022, 305, 135151. 35654232 10.1016/j.chemosphere.2022.135151

