# Supplementary material for: Superwettable‐Membrane‐Assisted Extraction and Separation as a General Method for Removal of Organic Pollutants From Water
Source: Adv Sci (Weinh). 2025 Oct 6;13(2):e15526. doi: 10.1002/advs.202515526 (PMC12786371; doi:10.1002/advs.202515526)
Supplement: Supplementary file 1 — Supporting Information [file ADVS-13-e15526-s001.pdf]

Supporting Information

Superwettable-membrane-assisted extraction and separation as a general method for removal of organic pollutants from water

Deqi Wang, Ting Zhang, Fan Min, Yifeng Gao, Jiaming Zhu, Ganhua Xie, and Zonglin Chu**

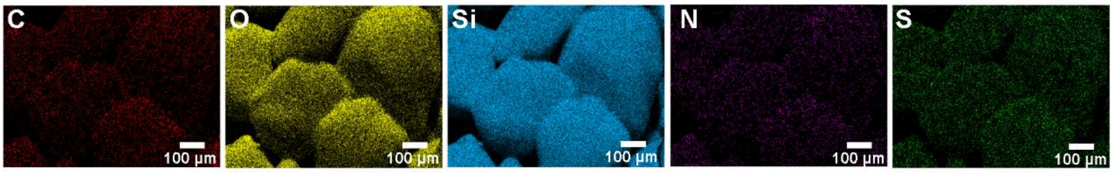

Figure S1. EDS mapping of sulfobetaine-modified G2 glass membrane.

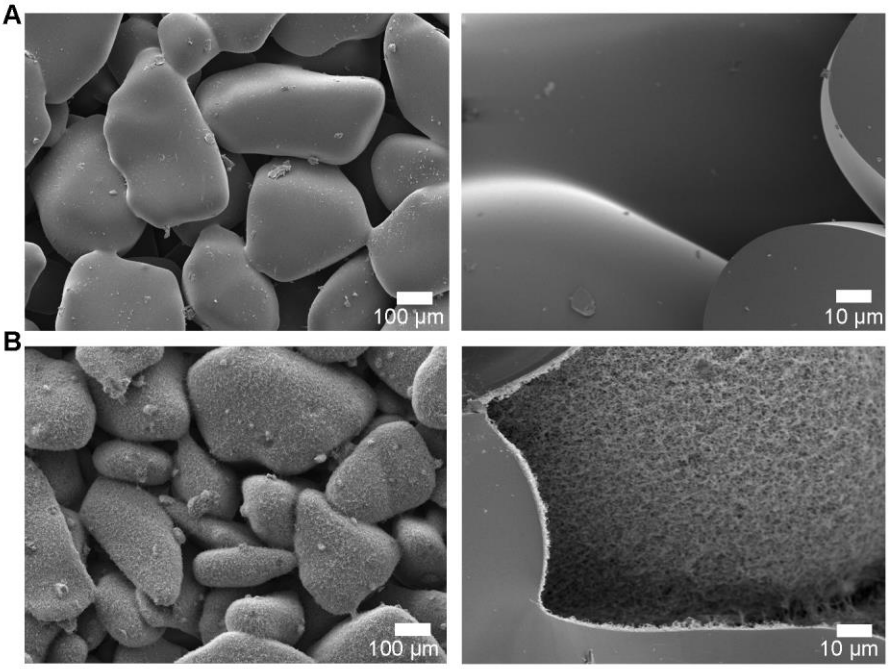

Figure S2. SEM images of (A) unmodified G2 glass membrane and (B) sulfobetaine-modified G2 glass membrane. Column 1 is the top views at lower magnifications, respectively. Column 2 represents the cross-section views of the materials.

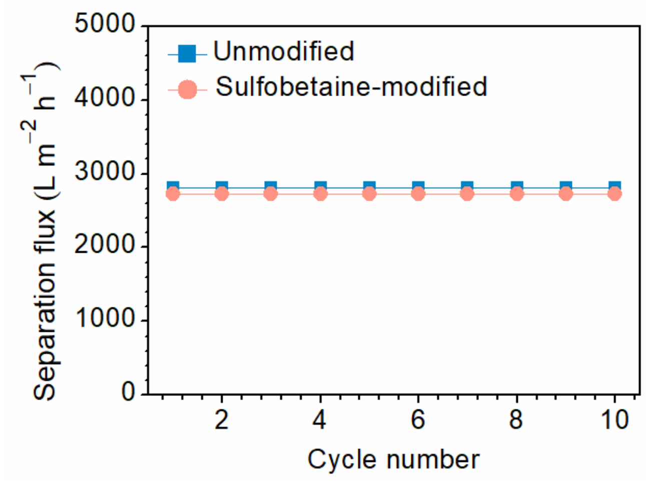

Figure S3. Separation flux of unmodified G2 glass membrane and sulfobetaine-modified G2 glass membrane for separation of pure water during 10 separation cycles (each 50 mL water was used as a cycle).

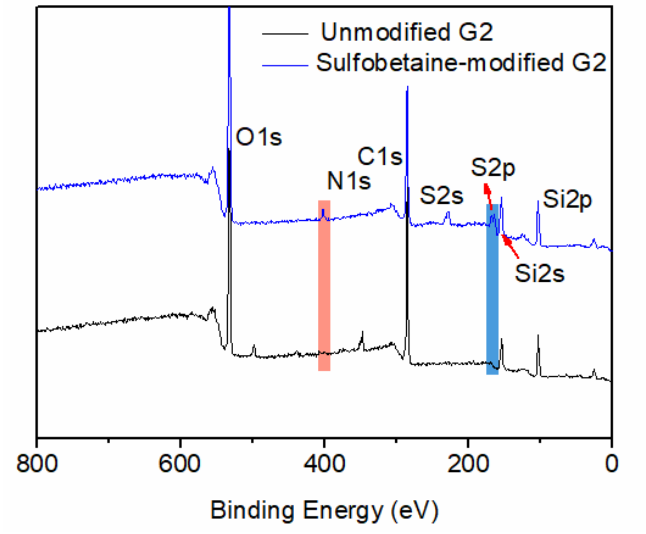

Figure S4. XPS survey spectra of the unmodified G2 membrane and sulfobetaine-modified G2 membrane.

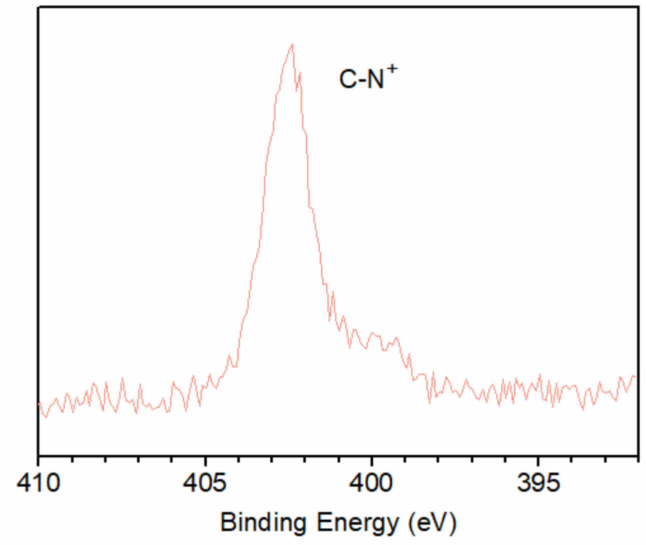

Figure S5. High-resolution XPS of the N 1s for sulfobetaine-modified G2 membrane.

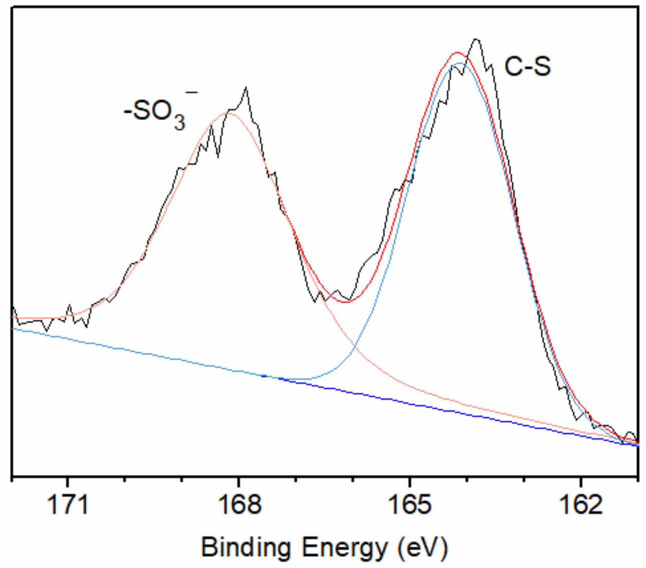

Figure S6. High-resolution XPS of the S 2p for sulfobetaine-modified G2 membrane.

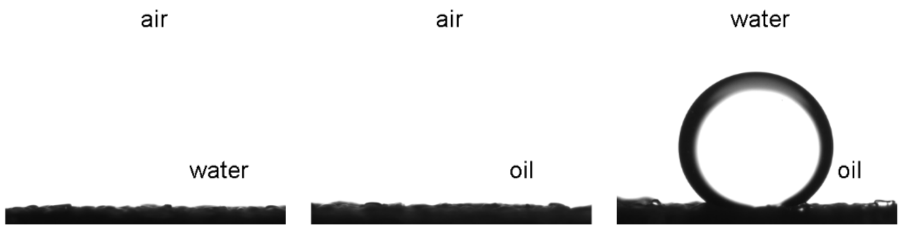

Figure S7. θ_W, θ_O and θ_{OW} for unmodified glass membrane.

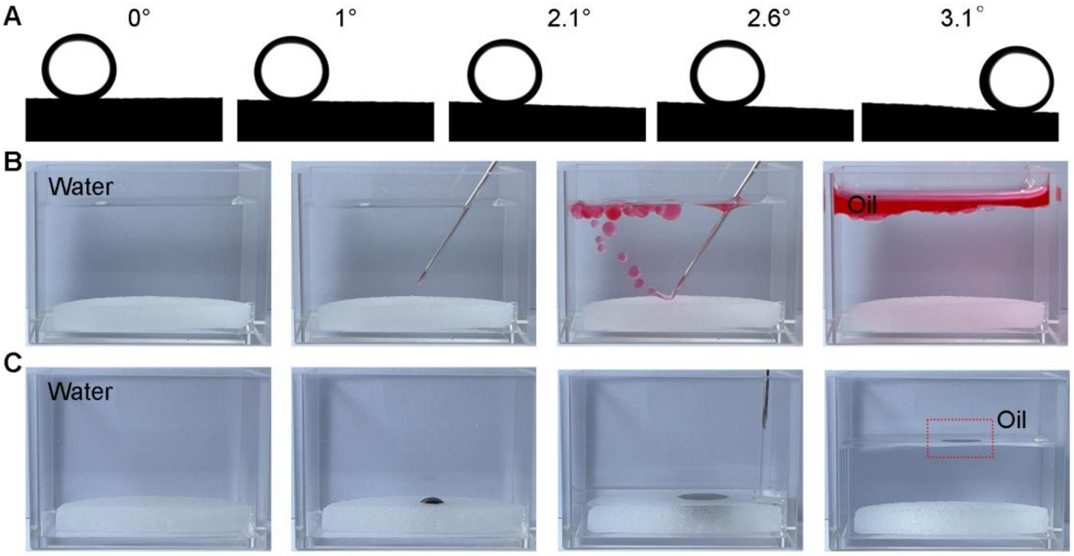

Figure S8. Underwater oil-adhesion resistance, and self-cleaning characteristic. (A) Series of snapshots showing underwater oil sliding angle of sulfobetaine-modified glass membrane. Oil was 1,2-dichloroethane. (B) A stream of low-density oil droplets was injected on the surface of the sulfobetaine-modified glass membrane. Oil was petroleum ether (dyed with oil red O). (C) Underwater self-cleaning properties of pre-wetted sulfobetaine-modified membrane surfaces contaminated with crude oil.

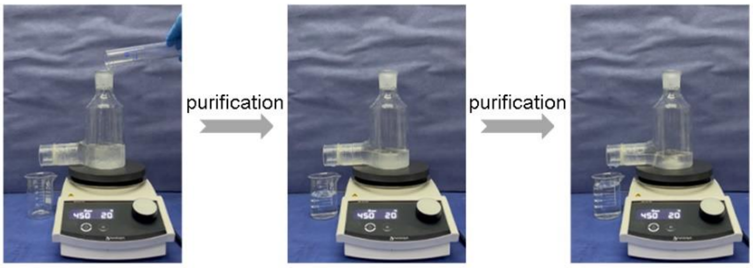

Figure S9. Extraction and separation of 100 ppm BPA.

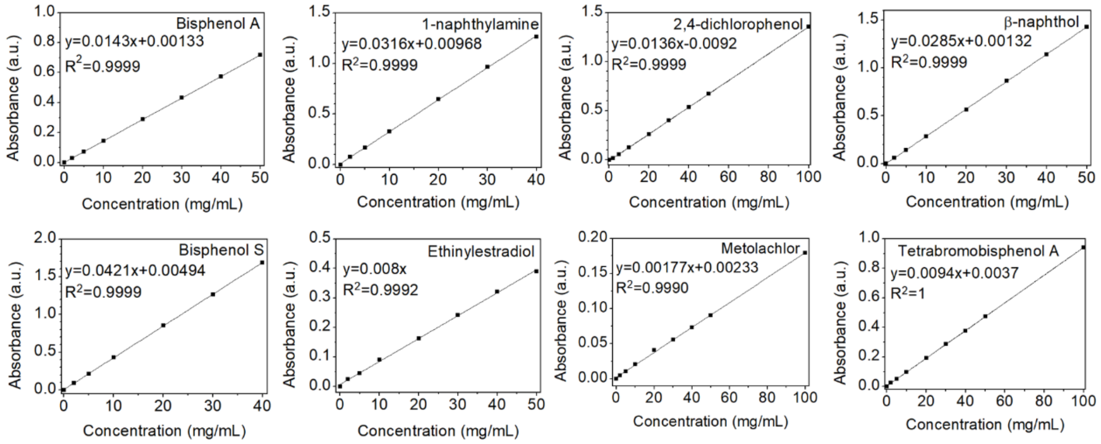

Figure S10. Standard curves for various organic pollutants. The organic pollutants were dissolved in a solution of 25 wt% of ethanol and water.

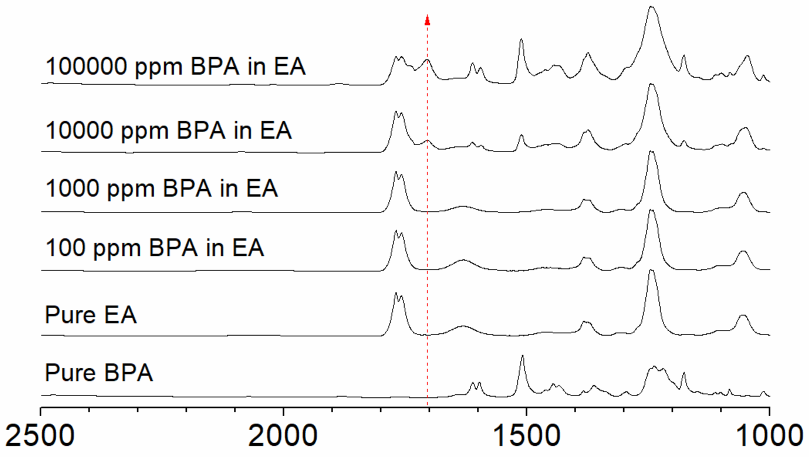

Figure S11. FTIR spectra of ethyl acetate (EA) at different concentrations of BPA.

Table S1. Partition coefficient of bisphenol A in different extractants and the removal efficiency of bisphenol A by different extractants.

Compound	Partition coefficient	Removal efficiency (%)
Ethyl acetate	480.9±82.6	99.8±0.04
Diethyl Ether	203.2±16	99.5±0.04
Ethyl butyrate	138.0±14.6	99.3±0.08
Butyl acetate	112.8±25.1	99.1±0.2
Butyl butyrate	76.4±10.3	98.7±0.2
Dichloromethane	36.1±2.6	97.3±0.2
Toluene	7.7±2.5	87.9±3.0
Petroleum ether	0.6±0.1	35.6±5.0

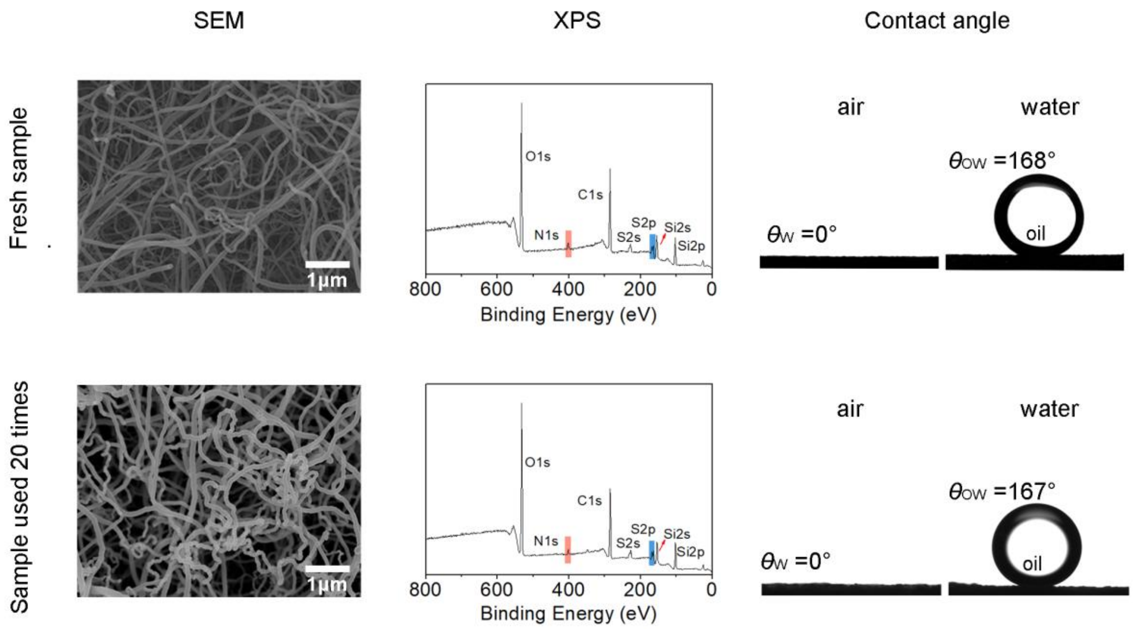

Figure S12. Comparisons between the fresh sample and the sample used 20 times by SEM, XPS analysis, and contact angle.

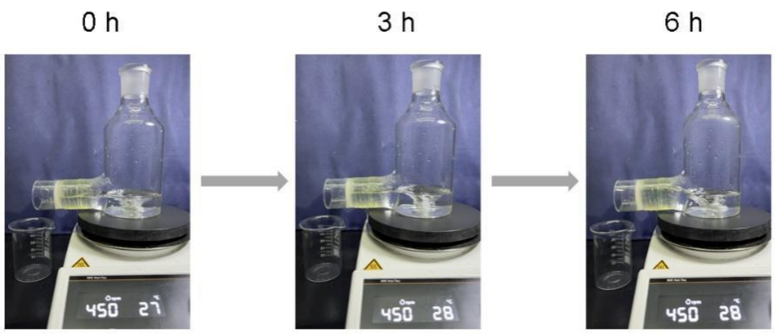

Figure S13. Sulfobetaine-modified G2 glass membrane pre-wetted with water resist oil penetration for a long period of time.

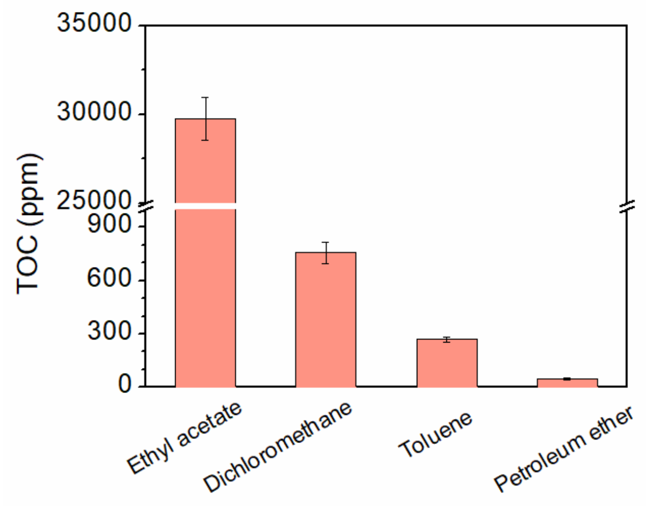

Figure S14. TOC values of filtrate at each stage after multi-stage extraction and separation of ethyl acetate-water mixture.

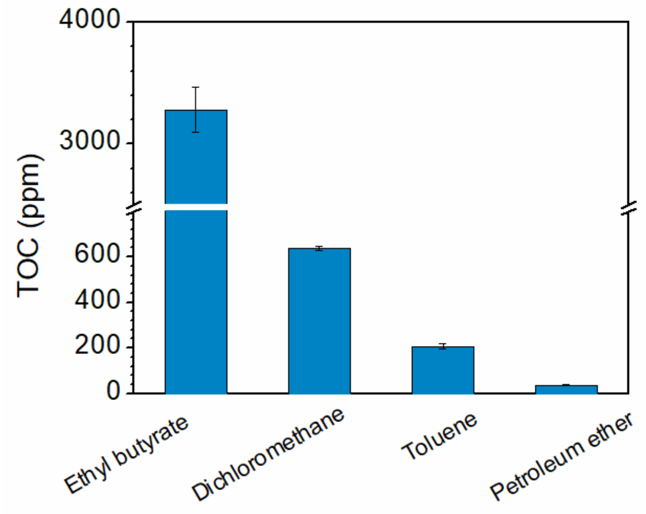

Figure S15. TOC values of filtrate at each stage after multi-stage extraction and separation of ethyl butyrate-water mixture.

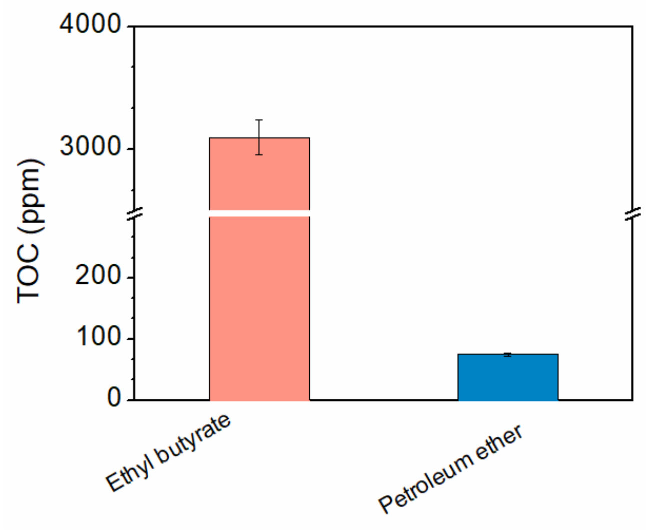

Figure S16. TOC values of filtrate at each stage after multi-stage extraction and separation of 100 ppm BPA.

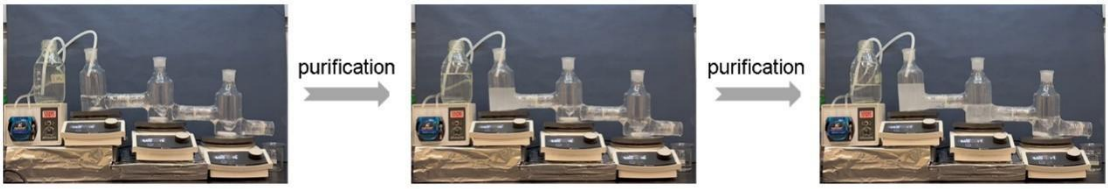

Figure S17. Home-made multi-stage extraction and separation purification system for the purification of organic pollutants.

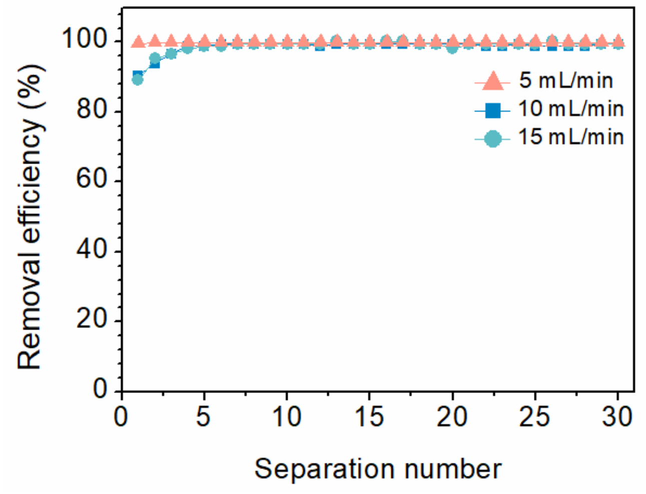

Figure S18. Removal efficiency for the separation of 100 ppm bisphenol A during 30 times continuous extraction and separation at a peristaltic pump rate of 5, 10 and15 mL min^{-1}, respectively.

Table S2. Comparison of individual liquid-liquid extraction, membrane separation, and the novel superwettable-membrane-assisted extraction and separation technology developed in this work for the removal of 100 ppm BPA from water.

Entry	Removal efficiency (%)	TOC of filtrate (ppm)
Individual liquid-liquid extraction	99.28	3400
Individual membrane separation	0	78
Superwettable-membrane-assisted extraction and separation	99.93	38

[a] liquid-liquid extraction performed with a separatory funnel using ethyl butyrate as the extractant.
[b] direct separation using a G2 glass filter after surface modification.
[c] the novel technology developed in this work.

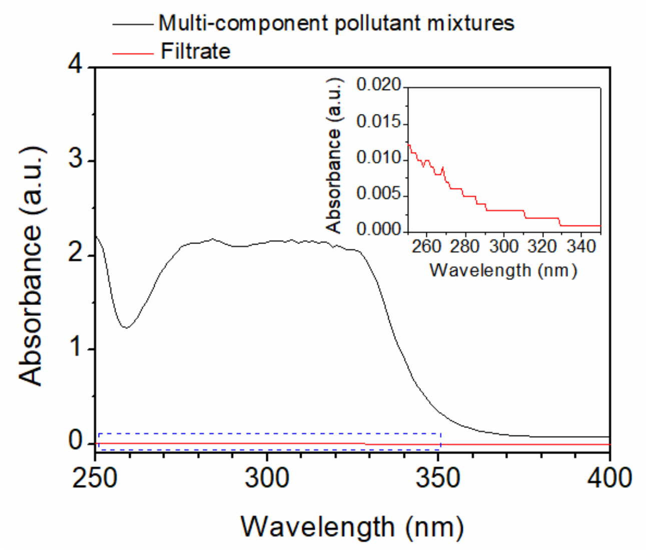

Figure S19. The UV-Vis spectra of 100 ppm multi-component pollutant mixtures (bisphenol A, 1-naphthylamine, tetrabromobisphenol A, ethinyl oestradiol and metolachlor) before and after multi-stage extraction and separation.

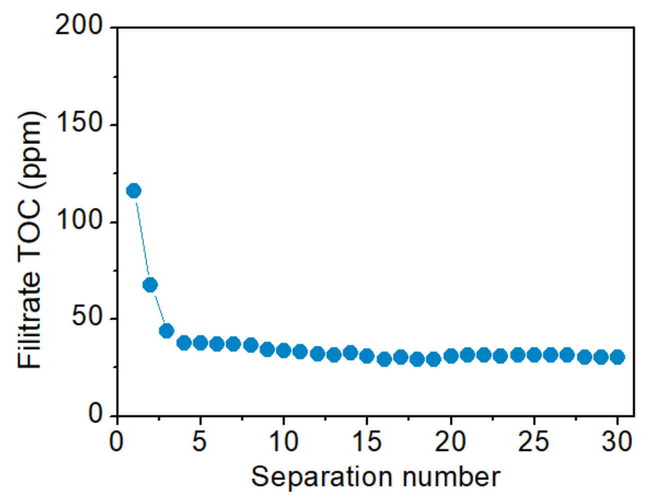

Figure S20. TOC for the separation of 100 ppm multi-component pollutant mixtures during 30 times continuous extraction and separation.
